# Case report of exercise and statin-fibrate combination therapy-caused myopathy in a patient with metabolic syndrome: contradictions between the two main therapeutic pathways

**DOI:** 10.1186/1756-0500-6-52

**Published:** 2013-02-06

**Authors:** Andrea László, László Kalabay, János Nemcsik

**Affiliations:** 1 Department of Family Medicine, Semmelweis University, Kútvölgyi str. 4, Budapest, 1125, Hungary

**Keywords:** Metabolic syndrome, Exercise, Statin-fibrate combination therapy, Myopathy, Creatine kinase

## Abstract

**Background:**

Lifestyle modifications including exercise are beneficial and fundamentally part of the therapy of metabolic syndrome, although in most of the cases medical interventions are also required to reach the target values in the laboratory parameters. Statin and fibrate combination therapy is considered to be safe and effective in dyslipidaemia and metabolic syndrome. However, increased physical activity can enhance the statin and fibrate-associated myopathy. Myositis and the rare but life-threatening rhabdomyolysis are causing a conflict between exercise and statin-fibrate therapy, which is yet to be resolved.

**Case presentation:**

We present a case of a 43-year-old Caucasian man with metabolic syndrome who had the side-effect of exercise and drug-associated myositis. The patient had only transient moderate complaints and rhabdomyolysis could be avoided with the one-month creatine kinase control, a test which is not recommended routinely by the new guidelines.

**Conclusions:**

We would like to turn the spotlight on the possible complications of statin-fibrate therapy and exercise, when strict follow-up is recommended. In this condition high number of patients can be affected and the responsibility of general practitioners is accentuated.

## Background

Cardiovascular disease is the leading cause of death in developed countries. In adults besides body mass index above 30 kg/m^2^ or elevated waist circumference, the clustered presence of 2 or more cardiovascular risk factors from among high triglyceride and fasting blood glucose levels, low high-density lipoprotein cholesterol (HDL-C), raised blood pressure and the presence of treated hypertension or type 2 diabetes has been designated the “metabolic syndrome” (MetS) [1]. These components are the principal risk factors in the development of atherosclerosis and cardiovascular disease. Related to this high risk status, the treatment of patients with MetS is an important task of the basic healthcare.

There is substantial evidence that regular exercise has beneficial effects in physical and mental health and contributes to the primary and secondary prevention of cardiovascular and several chronic diseases, like breast and colon cancer or diabetes [2]. Exercise improves a variety of lipid and lipoprotein variables [3] and is a fundamental part of the therapy of MetS.

The alternative of the lifestyle modifications is the use of tablets. For the correction of lipid disorders in MetS, recommendations, like the recent European guideline (European Society of Cardiology and the European Atherosclerosis Society (ESC/EAS 2011) [4]) aims a certain level of serum lipids. Not only statin or fibrate treatment, but their combination may be also required to achieve target lipid levels (cholesterol, LDL-C, triglycerides, HDL-C), which appears to confer additional cardiovascular risk – compared with LDL-C alone [5]. The combination therapy is especially recommended in MetS and diabetes with the administration of fenofibrate besides the statins [4].

Although the use of statins considered to be safe, some studies suggest that the frequency of statin myopathy is between 9% and 20% [6-8]. If muscle symptoms are accompanied by the elevation of creatine kinase (CK) levels, the condition is known as myositis [9-11]. Rhabdomyolysis is the most serious side-effect of the statin therapy, where renal insufficiency is present, as well. However, in some studies this side effect was found to be rare (affecting 0,1% of statin users) [12-15], and moreover no cases of drug-related rhabdomyolysis were reported in some follow-up studies [16,17]. Statins combined with fibrate also increase the incidence of statin-induced myopathies [11,18-20], suggested a 5.5-fold greater elevation compared with statin use alone [21].

In addition, physical exercise can also influence the occurrence of myopathy in patients using statins and fibrates. Muscle complaints may affect 25% of the statin users who exercise [22], and the prevalence of this phenomenon is 75% among athletes who take the drug [23].

Although the new ESC guideline on dyslipidaemias is expansively engaged in lifestyle modifications, it does not focus on the problem of the elevated risk of myopathy during statin-fibrate therapy and exercise [4]. This condition is not mentioned as a recommended reason for periodic CK control [4].

In our case report we present a patient with MetS who took statin and fibrate therapy and started parallel exercise leading to myositis with moderate symptoms but excessive CK elevation.

## Case presentation

On routine laboratory examination dyslipidaemia was found in a 43-year-old man without symptoms, who presented in our general practitioner praxis in October 2011. The patient was obese (body mass index 31.7 kg/m^2^, waist circumference 104 cm), with the history of 25 pack-year smoking and 2 units (20 ml) of alcohol intake per week. His hypertension started 10 years ago and was controlled with 2.5 mg nebivolol per day. He was a blue-collar worker in a storehouse, and sometimes did manual work (not every day and not exhausting).

His values were: total cholesterol 277.7 mg/dL, triglycerides 448.2 mg/dL, HDL-C 36.7 mg/dl, fasting plasma glucose level was 5.68 mmol/L. LDL-C could not have been evaluated, because the elevated serum triglyceride level disturbed the measurement. Based on data above, the patient fulfilled the requirements of MetS (central obesity, raised serum triglycerides, reduced HDL-C, treated hypertension and upper range of normal fasting glucose level were present). Other parameters, like creatine kinase (CK), aspartate transaminase (AST), alanine transaminase (ALT) and gamma glutamyl transpeptidase (GGT) were in the normal range (117 U/l, 26 U/l, 23 U/l and 36 U/l, respectively). Renal function (blood urea nitrogen: 7.6 mmol/l, serum creatinine: 97 μmol/L, glomerular filtration rate: >60 ml/min) was also normal. Past medical history included laparoscopic cholecystectomy in his 30s. From family history hypertension, hypercholesterinaemia and obesity of both parents must be mentioned. None of them had any side effect for drug administration (both of them took statins).

After informing the patient about his elevated cardiovascular risk, he ensured us to cooperate regarding taking the prescribed medications (except for aspirin, because of bad family experience). About lifestyle modifications he did not see possibilities for exercise because of his exhausting job. On the other hand, about diet he said, that he tried to decrease the calorie intake long ago, but because of his manual work he often felt hungry and required extra meals. He promised to keep this kind of diet in the future as well, which apparently was not enough. Due to the high triglycerides and lack of lifestyle modification possibilities, 160 mg fenofibrate per day was applied. The patient was also informed about the possible side-effects of the lipid-lowering therapy. Because of smoking, varenicline therapy was prescribed as well, as the patient previously failed to give up smoking with lifestyle modifications and the use of nicotine patch.

The patient came for one-month control without any symptoms, and mentioned that he had begun taking varenicline only one week earlier. Laboratory parameters revealed the efficacy of the therapy. Serum triglycerides were reduced, but remained above the target level (349 mg/dL), CK was in the normal range (121 U/l), but cholesterol showed further increase (310.5 mg/dL). Taking the lipid levels and the high cardiovascular risk of the patient due to MetS into consideration, 20 mg atorvastatin per day was added to the therapy. Similarly, a control laboratory test was set one month later.

The patient was also asymptomatic at the second control. This time both the values of triglycerides and cholesterol showed improvement, 154.1 and 249.8 mg/dL, respectively. However, in the CK level a more than 150-fold elevation was observed (18979 U/l). Kept on asking the possible symptoms, the patient told us that he had changed his mind about exercise and one week ago he bought body-building equipment. After he worked out with all kind of dumb-bells, he had moderate pain and stiff feeling everywhere in his chest and in both arms and legs and dark urine was observed parallel. The pain lasted for three days, than the symptoms disappeared. At the time of the control he had no muscle complaints.

He had stopped taking varenicline after 2 weeks, and had taken the three types of medicines together (statin, fibrate and varenicline) only one week long. He did not smoke since then. The irregular moderate alcohol consumption was continued during taking the prescribed medications, as well.

The findings of the physical examinations were normal. The electrocardiography did not show any relevant pathological abnormalities. Based on these data, the patient was sent to the emergency care unit of the territorial hospital with the diagnosis of rhabdomyolysis. He was admitted to the Department of Cardiology.

In the hospital on the day of admission in blood test results ALT (131.5 U/l) and AST (247.7 U/l) were elevated, while GGT remained in the normal range (38 U/l). The lactate dehydrogenase (LDH) level was also increased, 463 U/l. Statin and fibrate medications were stopped, and altogether 2 litres of Salsol infusion was administered. On the next days CK showed decreasing tendency (5134, 1823 U/l), and reduction was observed in one of the levels of liver transaminases (AST: 131.3; ALT: 95.5 U/l), and in LDH (386 U/l). Serum cholesterol level remained elevated (257.2 mg/dL), triglycerides reached the normal range (151.5 mg/dL). Kidney function parameters did not show any abnormalities, and the thyroid function test was also normal (thyroid-stimulating hormone 1.42 μU/ml). As the patient remained asymptomatic, and the laboratory controls indicated improvement, he was discharged following 2 days of hospitalization. Strict alcohol abstinence, 180 grams/day carbohydrate diet with low fat and salt intake were recommended at emission, completed with fish oil capsules and dietary sources of omega-3 fatty acids. Future contraindication of statin-fibrate combination therapy was stated, and laboratory control was advised in two days.

The control showed further improvement in the levels of transaminases, CK and LDH, but they were still moderately elevated (AST 58.8 U/l, ALT 72.1 U/l, CK 526 U/l, LDH 417 U/l). One month later the laboratory control was repeated in our praxis: CK and transaminases were normal, dyslipidaemia was reduced (total cholesterol and triglycerides levels were 238.3 and 216.1 mg/dL, respectively). Since that time the patient has been asymptomatic, keeps diet, exercises regularly (with no marked CK elevation), takes 1 gram omega-3 fatty acid per day, and thanks to the varenicline therapy he does not smoke.

The diagnosis of drug-induced myositis was reported to the Hungarian National Institute of Pharmacy.

According to the recommendation of ESC/EASC guideline for the management of dyslipidaemias, it is always appropriate to advise patients to engage in regular physical exercise of moderate intensity [24]. Lifestyle modification is recommended as the first step treating MetS, as well [4]. In fact, physical activity is one of the most cost-effective ways to prevent cardiovascular diseases.

However, lifestyle interventions considered being difficult to be maintained, and drugs are frequently prescribed as it is influenced by pharmaceutical industry pressure, as well [25]. Moreover, statin therapy has been suggested to be a fundamental tool of the prevention of coronary artery disease [26]. There is a growing concern that this total focus on statin use may distract attention from simple lifestyle changes and also interfere with exercise [22,27].

In our patient fibrate and later statin-fibrate medications were initiated because of the limited lifestyle changing possibilities. However, the patient changed his mind and started exercise which led to severe myositis but only moderate symptoms.

Although the patient had been already on combination therapy when the myositis started but the main cause of it was probably the atrovastatin as compared with gemfibrozil, fenofibrate were found to be less prone to make complications when used together with statins [28]. However, the pathophysiological role of fenofibrate or the exaggerating effect in the extent of CK elevation cannot be excluded.

Our case report shows the emerging problem of parallel application of exercise and statin-fibrate therapy. Although this problem is not new but still some questions are opened. It is not clear, what kind of exercise is safe and can be recommended besides statin-fibrate use. Especially prolonged exercise, like weight bearing and those which require eccentric muscular contractions (for example downhill treadmill walking) seem to exacerbate muscle injury [29-32]. So far no study has addressed the risk of exercise during statin and fibrate use or the possibly recommended type of gymnastics which are safe, but still beneficial in this condition.

Amongst other possible pathophysiological factors the moderate alcohol consumption of our patient probably did not influence the exercise-induced statin-myopathy as in low amounts (12.7-38.1 ml of ethanol per day in men) it was not found to be harmful [24].

Another question arises whether the statin-fibrate therapy-induced rhabdomyolysis could be connected to varenicline intake. The theoretical background of this interaction is based on the observation of Obrach et al., who found two metabolites in human excreta of varenicline: one of them is the N-carbamoylglucuronide, catalyzed by the UDP-glucuronosyltransferase-2B7 (UGT2B7) [33]. Fenofibrate is also glucuronidated by this enzyme [34]. Thus a varenicline-fenofibrate interaction is theoretically possible. In our case, 3 weeks after starting the fibrate therapy the patient began to take varenicline, and one week later atorvastatin. He took altogether a 2-week dose of varenicline where in the second week he took fenofibrate and atorvastatin, as well. The symptoms started two weeks after the stop of varenicline, so it probably had no role in the side effect.

Statin-fibrate combination should be prescribed with caution to patients who also receive other drugs that are metabolized through CYP 450. [4] Although the aromatic hydroxylation of nebivolol takes place in CYP 2D6 [35], a single nucleotid polymorphism of this type of cytochrome enzyme (the presence of CYP 2D6*4 allele) was also found to be associated with statin-induced muscle effects [36]. Although interaction between statins and nebivolol has not been published yet, nebivolol might have influenced the development of rhabdomyolysis in our patient. Unfortunately we did not have the opportunity to check the presence of this CYP 2D6*4 phenotype in our patient.

According to the guidelines, although pre-treatment CK level is required to be measured, routine monitoring of the enzyme without symptoms of myopathy/rhabdomyolysis, or risk factors is not necessary [4]. Furthermore, recent studies have suggested, that routine examinations for side effects are not cost-effective [10,37]. However, in our patient, who had no symptoms at the regular visits, the routine laboratory CK control indicated the severe myopathy and with the urgent intervention the development of acute renal failure and other severe complications could be prevented. Based on our case, the control of CK should also be added to lipid function control 4–6 weeks after the initiation of statin-fibrate combined therapy especially in those patients who do exercise parallel.

Finally, we must mention some emerging critical aspects of our medical attendance. With more impressions, the patient might have been convinced about the necessity of lifestyle modifications before the drug administrations. Unfortunately the patients' attitude and the insufficiency of time which can be expended for each patient in the office of a general practitioner sometimes limit our success. Secondly, fenofibrate was administered first instead of atrovastatin. This choice also can be debated, but we have chosen fibrate because of the more, than 3-fold higher triglyceride and the moderately elevated total cholesterol at the beginning. Third, unfortunately at the first blood test LDL-cholesterol could not have been evaluated because of the disturbing effect of raised triglyceride. Later at the control blood tests only total cholesterol was measured. LDL-cholesterol is an essential parameter of cardiovascular risk stratification and must be the part of the general laboratory protocol.

## Conclusions

In conclusion, although fenofibrate and statin combination therapy is supposed to be safe, myopathy, myositis and rhabdomyolysis still can develop. Exercise, as a part of lifestyle modifications can increase the risk of muscle side effects besides drug therapy providing a conflict between the two main therapeutic pathways of MetS. Further studies are required to define the types of exercise which are safe together with lipid lowering medications. The incidental interaction of nebivolol with statins or fibrates is also need to be clarified in the future. Moreover, also by asymptomatic patients the one-month CK control is important when exercise is present besides statin-fibrate therapy. In our case this test prevented the development of further serious complications.

## Consent

Written informed consent was obtained from the patient for publication of the Case report. A copy of a written consent is available for review by the Series Editor of this journal.

## Abbreviations

MetS: Metabolic syndrome; TG: Triglyceride; HDL-C: High-density lipoprotein cholesterol; ESC/EAS: European Society of Cardiology and the European Atherosclerosis Society; CK: Creatine kinase; AST: Aspartate transaminase; ALT: Alanine transaminases; GGT: Gamma glutamyl transpeptidase; LDH: Lactate dehydrogenase; CYP: Cytochrome P enzyme; GFR: Glomerular filtration rate.

## Competing interests

The authors declare that they have no competing interests.

## Authors’ contributions

AL has collected the required data of the patient and has written the manuscript. LK revised the manuscript critically for important intellectual content. JN treated and followed the patient and revised the manuscript. All authors read and approved the final manuscript.
